# Multimodal prediction of documented 1-year asthma exacerbation risk incorporating a static baseline CT digital-twin framework: a retrospective cohort study

**DOI:** 10.3389/fdgth.2026.1935315

**Published:** 2026-10-26

**Authors:** Mei Xie, Xiaofei Jin, Shaojin Wang, Hanchi Yu, Jindong Cheng, Shuni Wang, Yating Zhang, Xiaotong Guo, Jinxia Li, Hui Li, Hongyi Wang, Xiaoyan Yang

**Affiliations:** 1 Department of Geriatric and Special Needs Medicine Integrated Ward, General Hospital of Ningxia Medical University, Yinchuan, Ningxia, China; 2 Department of Pulmonary and Critical Care Medicine, General Hospital of Ningxia Medical University, Yinchuan, Ningxia, China; 3 Department of Pulmonary and Critical Care Medicine, Wuzhong People's Hospital, Wuzhong, Ningxia, China; 4 Department of Radiology, General Hospital of Ningxia Medical University, Yinchuan, Ningxia, China; 5 Department of Clinical Medicine, Chinese Academy of Medical Sciences and Peking Union Medical College, Beijing, China; 6 Department of Medical Sciences, Medical Laboratory of Innovation and Education Co., Ltd., Changsha, Hunan, China

**Keywords:** acute exacerbation, asthma, computed tomography, nomogram, risk prediction, static baseline CT digital-twin framework

## Abstract

**Background:**

To develop and conditionally evaluate a multimodal prediction model incorporating a static baseline CT digital-twin framework for documented 1-year asthma exacerbation risk.

**Methods:**

This retrospective single-center study analyzed 505 adults with asthma. Baseline three-dimensional CT reconstruction supplied quantitative features; the static baseline CT digital-twin framework did not simulate physiology or update dynamically. A LASSO-selected 10-variable logistic score was evaluated using nested domain comparisons, calibration, Brier score, and 1,000 bootstrap refits conditional on the selected predictor set. An available-variable clinical comparator was evaluated with and without CT features.

**Results:**

Seventy-two patients (14.3%) had a documented exacerbation. The final score had apparent AUC 0.880 (conditional bootstrap 95% CI, 0.836–0.917) and Brier score 0.086. Fixed-predictor bootstrap correction yielded AUC 0.856, calibration slope 0.875, intercept −0.182, and Brier score 0.094, without accounting for the original variable-selection step. CT increased AUC from 0.880 to 0.894 in the fully nested comparison (likelihood-ratio *P* = 0.070), and from 0.725 to 0.774 in the available-variable comparator (*P* < 0.001). These were apparent, exploratory comparisons, not external validation.

**Conclusion:**

The multimodal score showed conditional internal performance, with a modest CT increment in the fully nested comparison and a larger apparent increment in the restricted available-variable comparator. The CT contribution remains exploratory and comparator-dependent. External validation, full selection-aware validation, quantitative CT repeatability assessment, and prospective impact evaluation are required before clinical use.

## 1 Background

Asthma is a heterogeneous chronic airway disease that affects hundreds of millions of people worldwide and remains a major cause of preventable morbidity and health-care use (1). Acute exacerbations, commonly marked by worsening symptoms, reduced lung function, and the need for systemic corticosteroids, emergency care, or hospitalization, account for a disproportionate share of this burden (2, 3). Current risk assessment relies heavily on prior exacerbations, symptom control, medication use, and spirometry. These variables are clinically useful, but they may not fully capture the interacting inflammatory, physiologic, and structural changes that precede loss of asthma stability (4). More individualized tools are therefore needed to identify patients who may benefit from closer follow-up and earlier treatment review.

Prediction models based on electronic health records and machine-learning methods have shown that asthma exacerbation risk can be estimated from high-dimensional clinical data (5, 6). Similar work in other clinical prediction settings also suggests that flexible models may improve discrimination when the predictor space is complex (7). However, many existing models are driven mainly by administrative, treatment, or symptom variables and do not incorporate quantitative imaging markers of airway and vascular remodeling. This leaves an important gap: asthma exacerbation is a clinical event, but its risk is shaped by biological domains that include type 2 inflammation, systemic leukocyte activity, pulmonary mechanics, and CT-detectable structural changes.

Quantitative HRCT-derived structural and vascular features may complement clinical, physiologic, and inflammatory variables in asthma. Automated segmentation and three-dimensional reconstruction can yield airway, parenchymal, and vascular descriptors that are difficult to obtain by visual assessment alone (8–10).

We developed a multimodal prediction model incorporating a static baseline CT digital-twin framework that links patient-specific three-dimensional reconstruction to a probability score for documented 1-year acute exacerbation risk. The current implementation does not continuously update, simulate physiology, or provide bidirectional feedback. Its intended use is exploratory research, with incremental value requiring assessment in independent cohorts.

## 2 Methods

### 2.1 Patient data

This retrospective cohort study was approved by the Ethics Committee of the General Hospital of Ningxia Medical University (KYLL-2025-1724) and followed the Declaration of Helsinki. The committee waived informed consent for the entire retrospective study population. Records of patients treated between January 2020 and October 2024 were retrospectively reviewed using the 2025 GINA diagnostic criteria (11); this was retrospective diagnostic assessment, not use of a 2025 guideline during the original clinical encounters. Eligibility required age ≥18 years, bronchial asthma, baseline HRCT and key clinical assessments, and ascertainable one-year outcomes. Exclusions comprised interstitial lung disease or other major diffusion disorders, active malignancy, severe systemic infection, major cardiovascular events within 3 months, pregnancy or lactation, and unavailable outcomes or key predictors. Figure 1 presents 9,687 initial records, 6,741 adults screened, 6,236 aggregate exclusions and 505 analyzed participants, together with the verified exclusion categories. The analysis used the previously imputed 505-record table without additional imputation; performance estimates are conditional on this analysis table.

**Figure 1:**
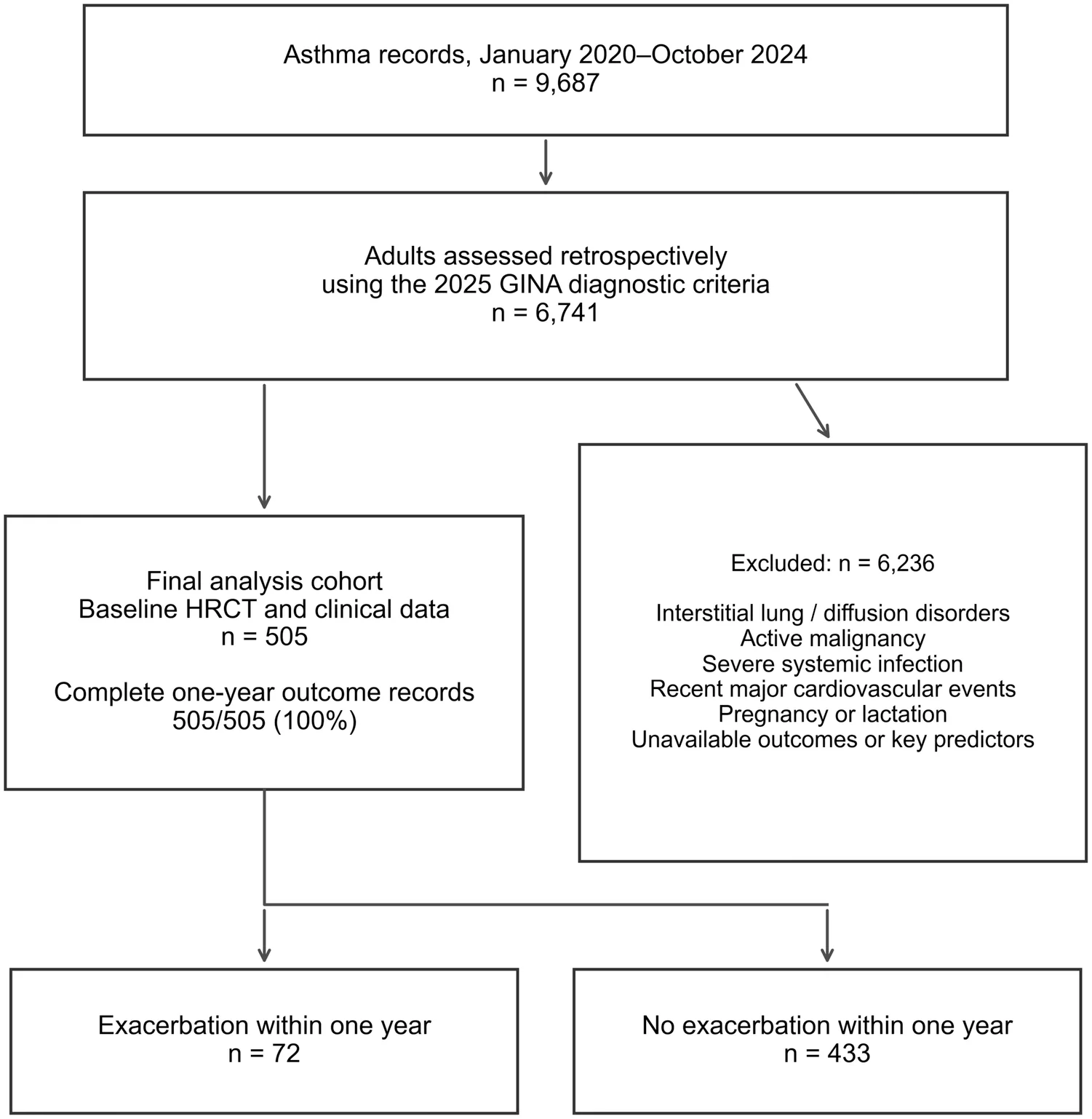
Retrospective cohort selection and one-year outcomes. Records from January 2020 to October 2024 were assessed retrospectively using the 2025 GINA diagnostic criteria: 9,687 initial records, 6,741 adults screened and 505 analyzed after 6,236 exclusions. Exclusion categories are shown without reason-specific counts. All 505 participants had complete one-year outcome records based on hospital records, telephone follow-up and a regional platform; 72 experienced an exacerbation and 433 did not. Telephone follow-up supplemented ascertainment of emergency visits and hospitalizations elsewhere. Informed consent was waived for the entire study population.

### 2.2 Acute exacerbation definition

Acute asthma exacerbation was defined according to GINA-based criteria as acute or subacute worsening requiring a change in treatment (11). The primary outcome was any exacerbation within one year after the index assessment requiring systemic corticosteroids for at least 3 days, an asthma-related emergency-department visit, or asthma-related hospitalization. One-year outcomes were ascertained using hospital electronic medical records, telephone follow-up and a regional health-information platform. All 505 participants had complete one-year outcome records (505/505, 100%); emergency visits or hospitalizations outside the study hospital were additionally ascertained by telephone follow-up.

### 2.3 Static baseline CT digital-twin framework

Baseline noncontrast HRCT was acquired during inspiration on a Brilliance 256 iCT scanner (Philips Healthcare), using 100–120 kVp and automatic tube-current modulation, with a reported tube current–time product of 150–200 mAs, 1-mm section thickness and 1-mm reconstruction interval. Reconstruction used the vendor-designated standard lung kernels YB and B, with iDose level 4. The static baseline CT digital-twin framework generated a patient-specific baseline representation using FACT AI + -digitalLung V1.0 (Shenzhou Dexin Medical Imaging Technology Co., Ltd., Weinan, Shaanxi, China). The software segmented the tracheobronchial tree, lung parenchyma and pulmonary vasculature and extracted quantitative measurements (Figure 2; Supplementary Table S1). The framework links this baseline representation to a fixed prediction score, without physiological simulation, dynamic updating or bidirectional feedback. Two radiologists, each with 10 years of thoracic imaging experience and blinded to outcomes, jointly reviewed all 505 segmentation results. No case required manual correction (0/505, 0%). This joint quality-control review supports segmentation review but does not quantify inter-reader agreement, repeated-segmentation reproducibility or scan–rescan repeatability. The segmentation software was obtained under a commercial license. The supplier did not participate in study design, data analysis, interpretation of results or manuscript writing. Internal software resampling used 1 × 1 × 1 mm isotropic voxels. Voxel-level review of the extreme maximum venous-density values confirmed the recorded measurements, with no measurement error identified.

**Figure 2:**
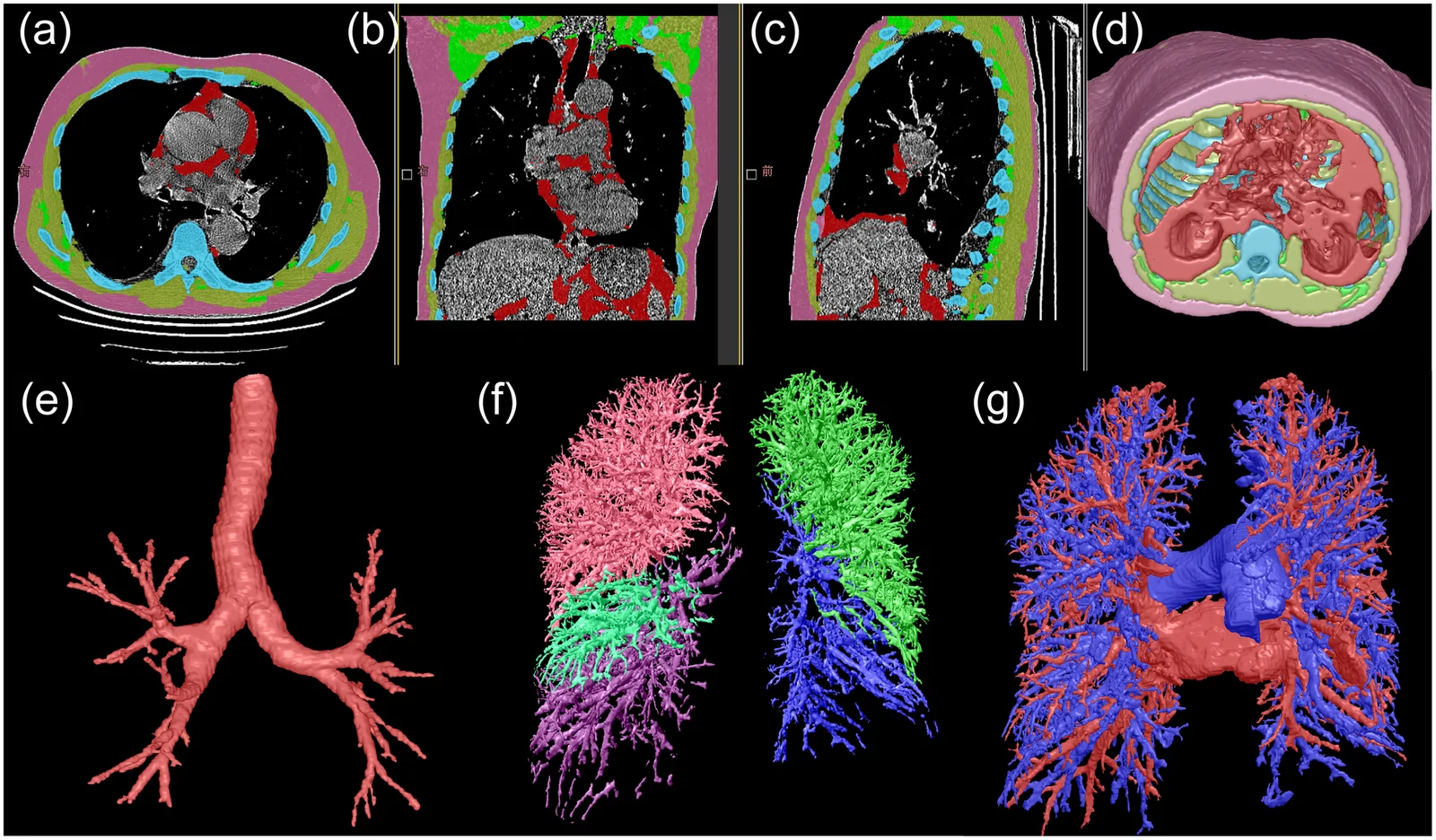
Static baseline CT digital-twin framework. (a-c) Representative axial, coronal and sagittal CT sections with multiclass segmentation. (d) Three-dimensional rendering of segmented thoracic structures. (e) Tracheobronchial tree reconstruction. (f) Lobe-color-coded three-dimensional reconstruction. (g) Pulmonary vascular network reconstruction. These patient-specific baseline representations were used for quantitative feature extraction and do not update dynamically or simulate physiology.

### 2.4 Data collection

Clinical and laboratory variables were collected from electronic records within 48 h of the index assessment when available. Laboratory fields included WBC, neutrophils, lymphocytes, MXD, eosinophils, basophils and total IgE. MXD was defined as the combined count of blood leukocyte subgroups other than neutrophils and lymphocytes, expressed in ×10^9/L.

### 2.5 Statistical analysis

Baseline characteristics were summarized by outcome group using Wilcoxon rank-sum, Pearson chi-square or Fisher exact tests. The original model was developed using LASSO with five-fold tuning and the lambda-1SE rule, followed by logistic regression using ten selected predictors. Original model development was performed automatically on the MSTAT platform using R 4.2.2 and platform-internal settings. The authors confirmed with the MSTAT platform administrator that the modeling parameter settings matched those documented for the Python selection implementation in Supplementary Methods S1. The fixed-predictor bootstrap validation was conditional on the ten selected predictors and did not repeat LASSO tuning or variable selection. An exploratory Python selection-resampling analysis is described separately in Supplementary Table S10. Nested domain models added laboratory, pulmonary-function and then CT variables to a clinical model (Supplementary Table S5). A separate available-variable clinical comparator partially overlapping with RESA predictor categories was fitted with and without CT (Supplementary Table S13) (12). Sex was coded Female=0 and Male=1; Yes/No fields were coded No = 0 and Yes=1. Smoking history was coded Yes=1 for current or former smokers (ever smokers) and No = 0 for never smokers; the comparison therefore represents ever versus never smoking. The final score used continuous variables on their recorded scales without additional outlier deletion or outcome-derived cut points. Paired 2,000-resample patient bootstraps held fitted predictions fixed to estimate conditional apparent AUC and Brier differences; these intervals do not account for model fitting or selection. Likelihood-ratio tests compared nested fits. Threshold and subgroup intervals likewise describe apparent performance conditional on the fitted score. Comparisons and coefficient *P*-values were exploratory and were not adjusted for selection or multiplicity.

## 3 Results

### 3.1 Patient characteristics

The study included 505 adults with asthma, of whom 72 (14.3%) had a documented acute exacerbation within 1 year. Baseline characteristics are shown in Table 1. The median age was 49 years (Q1, Q3: 37, 57), and 309 patients (61.2%) were female. Age, sex, weight, height, and BMI were broadly similar between patients with and without exacerbation.

**Table 1:** Patient demographics and baseline characteristics.

Characteristic	Acute Exacerbation			*p*-value
	Overall *N* = 505	No *N* = 433	Yes *N* = 72	
Age, years, Median (Q1, Q3)	49 (37, 57)	49 (36, 57)	51 (43, 61)	0.0641
Gender, *n* (%)				0.5912
Female	309 (61.2%)	267 (61.7%)	42 (58.3%)	
Male	196 (38.8%)	166 (38.3%)	30 (41.7%)	
Weight, kg, Median (Q1, Q3)	64 (57, 72)	65 (57, 72)	63 (56, 74)	0.9661
Height, cm, Median (Q1, Q3)	161 (156, 168)	161 (156, 168)	163 (154, 169)	0.8161
BMI, kg/m2, Median (Q1, Q3)	24.4 (22.0, 27.0)	24.5 (22.0, 27.0)	24.4 (22.2, 27.1)	0.9881
Smoking history, *n* (%)				0.0122
Never smokers (No)	55 (10.9%)	41 (9.5%)	14 (19.4%)	
Ever smokers (Yes)	450 (89.1%)	392 (90.5%)	58 (80.6%)	
VC%, Median (Q1, Q3)	100 (91, 110)	100 (92, 111)	94 (85, 103)	<0.0011
FVC%, Median (Q1, Q3)	101 (90, 113)	102 (91, 115)	92 (83, 106)	<0.0011
FEV1%, Median (Q1, Q3)	84 (69, 96)	85 (73, 96)	71 (56, 89)	<0.0011
FEV1/FVC, Median (Q1, Q3)	69 (60, 76)	70 (62, 76)	64 (57, 74)	0.0061
PEF%, Median (Q1, Q3)	85 (67, 100)	86 (70, 101)	67 (53, 93)	<0.0011
TLC%, Median (Q1, Q3)	109 (107, 111)	109 (107, 109)	113 (105, 135)	<0.0011
DLCO%, Median (Q1, Q3)	102 (102, 102)	102 (102, 102)	102 (91, 102)	<0.0011
Diagnosis, *n* (%)				<0.0012
Cough Variant Asthma	127 (25.1%)	121 (27.9%)	6 (8.3%)	
Other	378 (74.9%)	312 (72.1%)	66 (91.7%)	
Disease Duration Years, Median (Q1, Q3)	1.0 (0.0, 3.0)	1.0 (0.0, 2.0)	3.0 (1.0, 8.0)	<0.0011
ICS, *n* (%)				0.0832
No	141 (27.9%)	127 (29.3%)	14 (19.4%)	
Yes	364 (72.1%)	306 (70.7%)	58 (80.6%)	
LABA, *n* (%)				0.0282
No	138 (27.3%)	126 (29.1%)	12 (16.7%)	
Yes	367 (72.7%)	307 (70.9%)	60 (83.3%)	
LAMA, *n* (%)				0.3312
No	462 (91.5%)	394 (91.0%)	68 (94.4%)	
Yes	43 (8.5%)	39 (9.0%)	4 (5.6%)	
SABA, *n* (%)				0.6093
No	497 (98.4%)	425 (98.2%)	72 (100.0%)	
Yes	8 (1.6%)	8 (1.8%)	0 (0.0%)	
LTRA, *n* (%)				0.2012
No	407 (80.6%)	345 (79.7%)	62 (86.1%)	
Yes	98 (19.4%)	88 (20.3%)	10 (13.9%)	
Methylxanthines, *n* (%)				0.0132
No	437 (86.5%)	368 (85.0%)	69 (95.8%)	
Yes	68 (13.5%)	65 (15.0%)	3 (4.2%)	
OCS, *n* (%)				>0.9993
No	487 (96.4%)	417 (96.3%)	70 (97.2%)	
Yes	18 (3.6%)	16 (3.7%)	2 (2.8%)	
Antihistamines, *n* (%)				0.1453
No	490 (97.0%)	418 (96.5%)	72 (100.0%)	
Yes	15 (3.0%)	15 (3.5%)	0 (0.0%)	
Compound Methoxyphenamine, *n* (%)				0.4983
No	486 (96.2%)	415 (95.8%)	71 (98.6%)	
Yes	19 (3.8%)	18 (4.2%)	1 (1.4%)	
Rhinitis, *n* (%)				0.0542
No	351 (69.5%)	294 (67.9%)	57 (79.2%)	
Yes	154 (30.5%)	139 (32.1%)	15 (20.8%)	
COPD, *n* (%)				0.0042
No	434 (85.9%)	380 (87.8%)	54 (75.0%)	
Yes	71 (14.1%)	53 (12.2%)	18 (25.0%)	
Polyp Sinusitis, *n* (%)				0.0993
No	477 (94.5%)	412 (95.2%)	65 (90.3%)	
Yes	28 (5.5%)	21 (4.8%)	7 (9.7%)	
WBC, Median (Q1, Q3)	6.89 (6.69, 7.26)	6.89 (6.69, 6.89)	8.06 (6.73, 9.48)	<0.0011
NEUT, Median (Q1, Q3)	3.90 (3.63, 4.22)	3.90 (3.63, 3.90)	4.65 (3.63, 6.13)	<0.0011
LYM, Median (Q1, Q3)	2.00 (1.92, 2.17)	2.00 (2.00, 2.02)	2.00 (1.51, 2.53)	0.9351
MXD, Median (Q1, Q3)	0.48 (0.46, 0.50)	0.48 (0.46, 0.48)	0.56 (0.45, 0.70)	<0.0011
EOS, Median (Q1, Q3)	0.24 (0.19, 0.33)	0.24 (0.22, 0.24)	0.33 (0.08, 0.70)	0.0501
BASO, Median (Q1, Q3)	0.040 (0.030, 0.040)	0.040 (0.040, 0.040)	0.045 (0.030, 0.070)	0.0431
IgE, Median (Q1, Q3)	135 (135, 135)	135 (135, 135)	135 (134, 345)	0.0011

a Wilcoxon rank sum test.

b Pearson's Chi-squared test.

c Fisher's exact test.

BMI, Body Mass Index; COPD, Chronic Obstructive Pulmonary Disease; VC, Vital Capacity; FVC, Forced Vital Capacity; FEV1, Forced Expiratory Volume in 1 s; PEF, Peak Expiratory Flow; TLC, Total Lung Capacity; DLCO, Diffusing Capacity of the Lungs for Carbon Monoxide; ICS, Inhaled Corticosteroids; LABA, Long-Acting Beta2-Agonists; LAMA, Long-Acting Muscarinic Antagonists; SABA, Short-Acting Beta2-Agonists; LTRA, Leukotriene Receptor Antagonists; OCS, Oral Corticosteroids; WBC, White Blood Cell; NEUT, Neutrophil; LYM, Lymphocyte; MXD, combined count of blood leukocyte subgroups other than neutrophils and lymphocytes; EOS, Eosinophil; BASO, Basophil; IgE, Immunoglobulin E; Q1, Q3: First quartile (25th percentile) and third quartile (75th percentile). Smoking history: Yes denotes current or former smoking (ever smoking); No denotes never smoking.

Several clinical features differed by exacerbation status. Disease duration was longer among patients with exacerbation [median, 3.0 years (1.0, 8.0)] than among those without exacerbation [median, 1.0 year (0.0, 2.0); *p* < 0.001]. COPD was also more frequent in the exacerbation group (25.0% vs. 12.2%; *p* = 0.004). The distribution of asthma diagnosis category differed between groups (*p* < 0.001), with cough-variant asthma less common among patients who experienced exacerbation. Smoking-status distribution also differed (*p* = 0.012).

Patients with exacerbation had lower VC%, FVC%, FEV1%, FEV1/FVC, PEF% and DLCO%, and higher TLC% (Table 1).

LABA use was more frequent in patients with exacerbation (83.3% vs. 70.9%; *P* = 0.028), whereas methylxanthine use was less frequent (4.2% vs. 15.0%; *P* = 0.013). The ICS comparison was not statistically significant (*P* = 0.083). These observational medication differences may reflect confounding by indication and cannot be interpreted as treatment effects.

Inflammatory and immunologic markers also differed by outcome. Patients with exacerbation had higher WBC, neutrophil count, MXD, basophil count, and IgE levels (Table 1). Eosinophil count showed a borderline difference (*p* = 0.050), whereas lymphocyte count was similar between groups.

### 3.2 Predictive model development

LASSO retained disease duration, VC%, FVC%, TLC%, DLCO%, WBC, MXD, IgE, artery volume and maximum venous density for the final score (Table 2; Figure 3). Supplementary Table S1 defines the CT feature families, and Supplementary Table S15 reports the complete penalized coefficient vector. Exploratory selection frequencies from an alternative Python implementation are provided in Supplementary Table S10; they are not post-selection significance tests or exact replication of the original R workflow.

**Table 2:** Nonzero coefficients of the LASSO-selected score.

Variable	LASSO coefficient
(Intercept)	−5.84961988929
Disease Duration Years	0.04002332850
VC percent	−0.00297878502
FVC percent	−0.00417291507
TLC percent	0.02641285646
DLCO percent	−0.00196607009
WBC	0.06703868185
MXD	1.91750919618
IgE	0.00115869285
Artery Volume mL	0.00060838793
Vein Max Density HU	0.00008693638

Coefficients are the penalized LASSO estimates, not the unpenalized final prediction equation. VC, vital capacity; FVC, forced vital capacity; TLC, total lung capacity; DLCO, diffusing capacity for carbon monoxide; WBC, white blood cell count; MXD, combined count of blood leukocyte subgroups other than neutrophils and lymphocytes; IgE, immunoglobulin E; HU, Hounsfield unit. See Supplementary Table S9 for the original-scale prediction equation.

**Figure 3:**
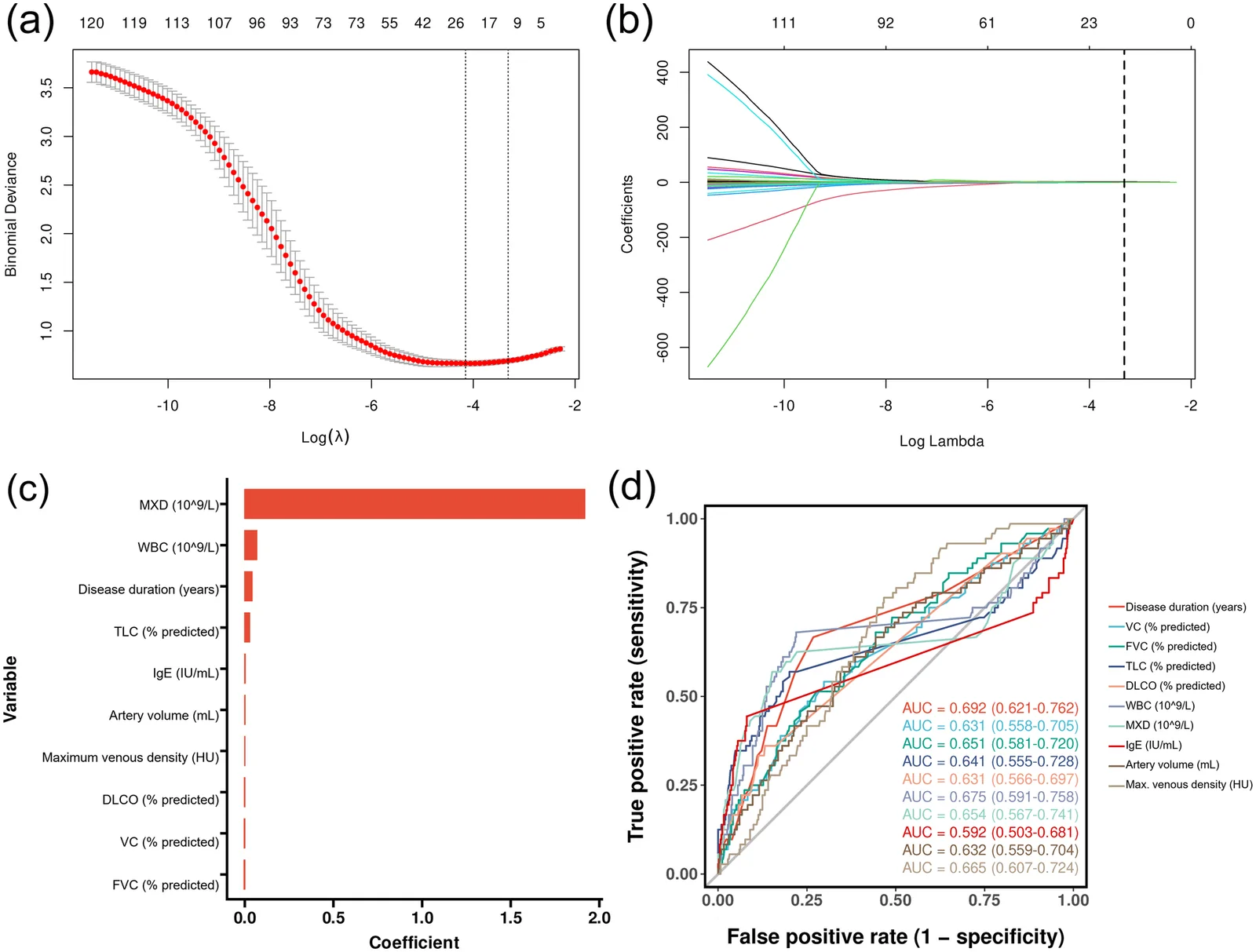
LASSO selection plots and single-predictor performance. (a) Tuning curve. (b) Coefficient path. (c) Selected coefficients. (d) Apparent single-predictor ROC curves. The exploratory selection-resampling analysis in Supplementary Table S10 uses a separate implementation.

Individual predictors had only modest discrimination (Figure 3D). The highest single-variable AUC was observed for disease duration (AUC, 0.692; 95% CI, 0.621–0.762). Other selected variables had AUCs ranging from 0.592 for IgE to 0.675 for WBC. No single predictor achieved an AUC above 0.700, indicating that each variable alone was insufficient for reliable risk stratification.

The final score is a prediction model, not a hypothesis-testing model. Supplementary Table S9 reports the original-scale intercept and ten coefficients. Disease duration, TLC%, WBC, MXD, IgE, artery volume and maximum venous density had positive fitted coefficients, while VC%, FVC% and DLCO% had negative coefficients. Nominal coefficient *P*-values are descriptive and not adjusted for selection or multiplicity. Univariable and multivariable logistic regression results are presented in Table 3.

**Table 3:** Univariable and multivariable logistic regression results using clinically interpretable predictor scales.

Characteristic	N	Event N	OR	95% CI	*p*-value
Univariate					
Disease duration, per 1 year	505	72	1.09	1.05, 1.13	<0.001
VC%, per 10% predicted	505	72	0.73	0.62, 0.86	<0.001
FVC%, per 10% predicted	505	72	0.75	0.65, 0.87	<0.001
TLC%, per 10% predicted	505	72	1.59	1.34, 1.88	<0.001
DLCO%, per 10% predicted	505	72	0.68	0.58, 0.81	<0.001
WBC, per 1 × 10^9/L	505	72	1.34	1.20, 1.49	<0.001
MXD, per 0.1 × 10^9/L	505	72	1.53	1.32, 1.79	<0.001
IgE, per 100 IU/mL	505	72	1.29	1.15, 1.45	<0.001
Artery volume, per 10 mL	505	72	1.20	1.10, 1.31	<0.001
Maximum venous density, per 100 HU	505	72	1.07	1.03, 1.11	<0.001
Multivariate					
Disease duration, per 1 year	505	72	1.07	1.03, 1.12	0.002
VC%, per 10% predicted	505	72	0.82	0.52, 1.29	0.393
FVC%, per 10% predicted	505	72	0.91	0.62, 1.35	0.651
TLC%, per 10% predicted	505	72	1.60	1.32, 1.95	<0.001
DLCO%, per 10% predicted	505	72	0.90	0.72, 1.12	0.328
WBC, per 1 × 10^9/L	505	72	1.14	0.97, 1.34	0.118
MXD, per 0.1 × 10^9/L	505	72	1.31	1.07, 1.61	0.009
IgE, per 100 IU/mL	505	72	1.25	1.11, 1.42	<0.001
Artery volume, per 10 mL	505	72	1.05	0.94, 1.17	0.416
Maximum venous density, per 100 HU	505	72	1.06	1.01, 1.12	0.019

OR, odds ratio; CI, confidence interval; VC, vital capacity; FVC, forced vital capacity; TLC, total lung capacity; DLCO, diffusing capacity of the lungs for carbon monoxide; WBC, white blood cell count; MXD, combined count of blood leukocyte subgroups other than neutrophils and lymphocytes; IgE, immunoglobulin E; HU, Hounsfield unit. ORs are expressed per clinically meaningful unit as specified in the variable labels.

All ten selected predictors were retained in the composite score regardless of their nominal multivariable *P*-values (Figure 4). The web-based calculator is available at https://zryh2023.shinyapps.io/dyn-nomapp/ as a research demonstration; it has not undergone external or prospective impact validation and should not guide individual treatment decisions. The webpage now displays a prominent warning that the model is intended for research use only and has not been externally validated.

**Figure 4:**
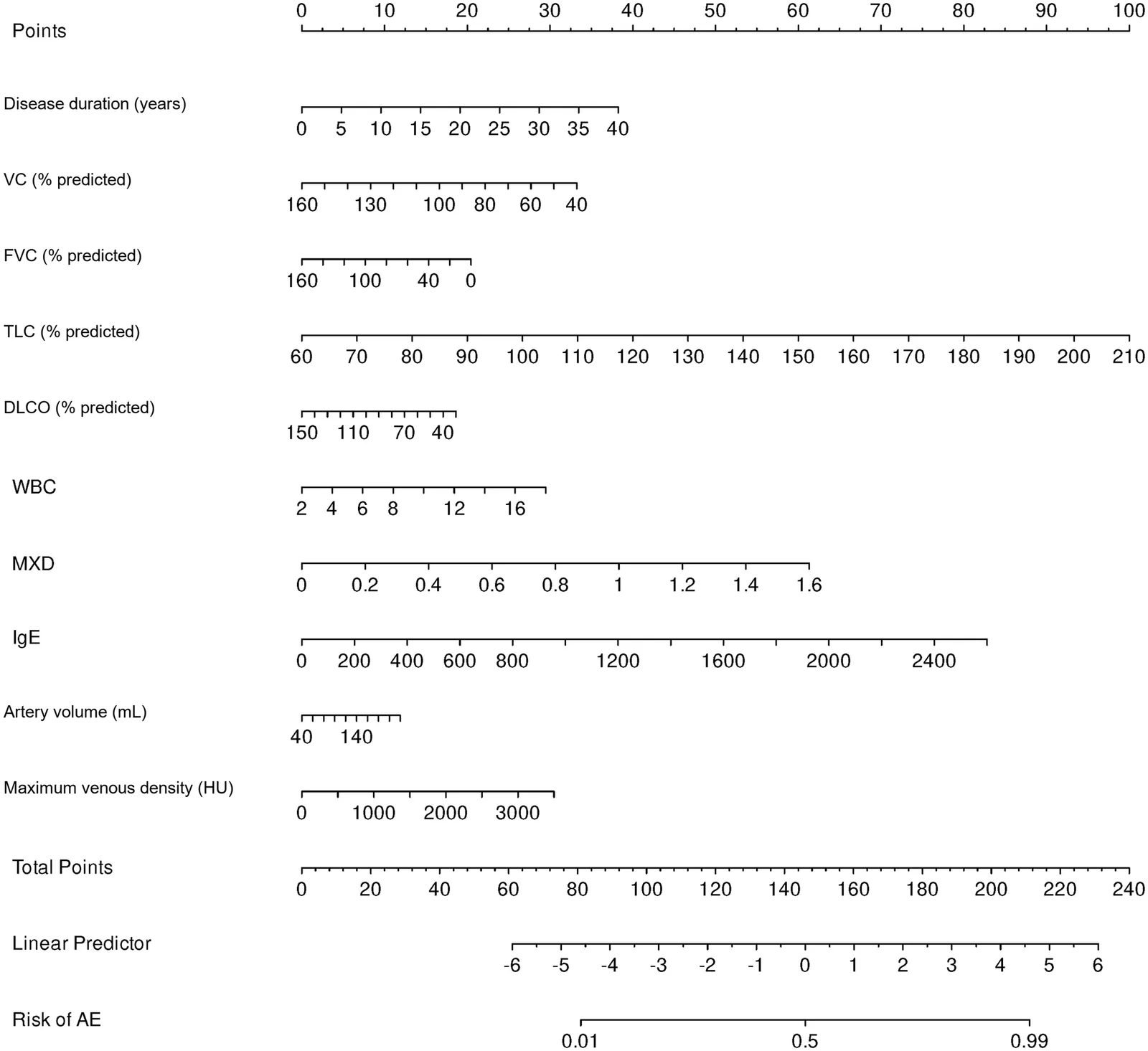
Multimodal nomogram for documented 1-year acute exacerbation risk. The displayed score uses the original-scale coefficients and intercept provided in Supplementary Table S9; quantitative CT variables are artery volume (mL) and maximum venous density (HU).

### 3.3 Predictive model validation

The final ten-predictor score had apparent AUC 0.880 (conditional bootstrap 95% CI, 0.836–0.917) and Brier score 0.086. Apparent discrimination, calibration and decision curves are shown in Figure 5. Nested-model apparent AUCs were 0.735 (clinical), 0.833 (clinical plus laboratory), 0.880 (plus pulmonary function), and 0.894 (plus CT); Brier scores were 0.113, 0.094, 0.084 and 0.083, respectively (Supplementary Table S5). The CT increment was 0.013 (conditional bootstrap 95% CI, −0.001 to 0.031; likelihood-ratio chi-square=5.32, 2 df, *P* = 0.070). In the available-variable comparator, AUC was 0.725 without CT and 0.774 with CT (difference 0.049; conditional bootstrap 95% CI, 0.008–0.092; likelihood-ratio chi-square=17.83, 2 df, *P* < 0.001). Brier score decreased from 0.111 to 0.107, but the improvement interval included zero (0.0039; 95% CI, −0.0018 to 0.0095; Supplementary Table S13). Fixed-predictor bootstrap correction yielded AUC 0.856, calibration slope 0.875, intercept −0.182 and Brier score 0.094 (Supplementary Table S6). Exploratory selection resampling showed mean bootstrap apparent AUC 0.963 and mean AUC 0.877 when those fitted models were evaluated on the original, overlapping cohort (Supplementary Table S10). Average precision was 0.600; the null Brier score was 0.122 and scaled Brier score 0.296. Five-fold summaries from the original analysis reported accuracy 0.858 versus a no-information rate of 0.857, kappa 0.293, sensitivity 0.332 and specificity 0.956 (Supplementary Tables S3–S4). At a threshold of 0.10, apparent sensitivity was 86.1% and specificity 76.2% (Table 4; Supplementary Table S12). CT-density and COPD sensitivity analyses are reported in Supplementary Table S11. Exploratory apparent subgroup ROC curves from the fixed ten-predictor score are shown in Figure 6.

**Figure 5:**
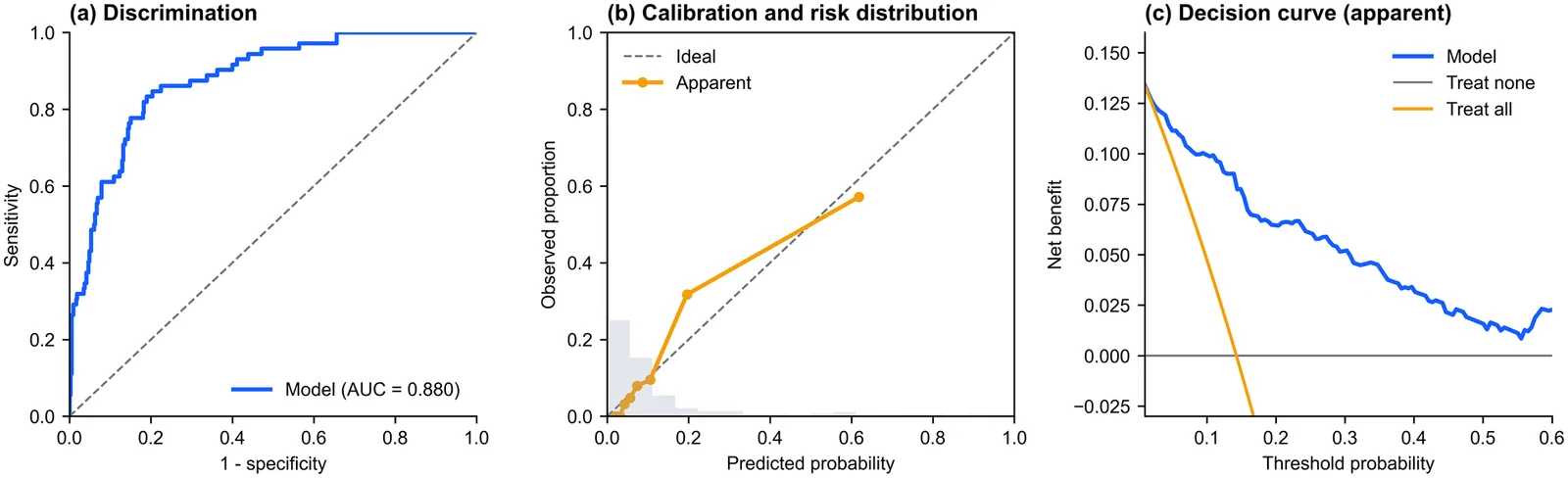
Apparent performance of the ten-predictor score. **(a)** ROC curve. **(b)** Quantile-binned calibration with the distribution of predicted risks; the curve describes in-sample agreement and is not an optimism-corrected calibration curve. **(c)** Apparent decision curves relative to treat-all and treat-none strategies; these do not establish clinical impact.

**Table 4:** Classification accuracy for prediction at different risk cutoff points for the model.

Risk threshold	Sensitivity (%)	Specificity (%)	PPV (%)	NPV (%)	Accuracy (%)	F1 score
≥0.0	100.0 (100.0–100.0)	0.0 (0.0–0.0)	14.3 (11.5–17.1)	NE	14.3 (11.5–17.1)	25.0 (20.6–29.3)
≥0.1	86.1 (77.4–93.7)	76.2 (71.8–80.0)	37.6 (30.5–44.3)	97.1 (95.2–98.7)	77.6 (74.1–81.3)	52.3 (44.6–59.5)
≥0.2	61.1 (48.1–72.0)	89.6 (86.9–92.5)	49.4 (38.4–60.0)	93.3 (90.8–95.6)	85.5 (82.6–88.3)	54.7 (43.9–64.6)
≥0.3	52.8 (40.4–63.9)	93.8 (91.4–96.0)	58.5 (45.8–70.9)	92.3 (89.6–94.4)	87.9 (84.8–90.7)	55.5 (44.5–65.3)
≥0.4	41.7 (30.5–53.3)	95.2 (93.0–97.0)	58.8 (45.7–72.0)	90.7 (87.8–93.4)	87.5 (84.4–90.3)	48.8 (37.4–59.2)
≥0.5	36.1 (25.5–47.3)	95.8 (93.9–97.7)	59.1 (44.7–74.4)	90.0 (87.4–92.9)	87.3 (84.4–90.3)	44.8 (33.2–55.9)
≥0.6	30.6 (20.6–41.5)	98.4 (97.2–99.5)	75.9 (59.3–90.6)	89.5 (86.7–92.1)	88.7 (86.0–91.3)	43.6 (31.1–54.9)
≥0.7	26.4 (17.1–37.5)	99.5 (98.8–100.0)	90.5 (76.5–100.0)	89.0 (86.5–91.8)	89.1 (86.3–91.7)	40.9 (28.4–53.7)
≥0.8	16.7 (8.7–26.1)	99.5 (98.8–100.0)	85.7 (66.7–100.0)	87.8 (85.0–90.5)	87.7 (85.0–90.3)	27.9 (15.6–40.8)
≥0.9	11.1 (4.7–19.0)	99.5 (98.8–100.0)	80.0 (50.0–100.0)	87.1 (84.0–89.8)	86.9 (83.9–89.6)	19.5 (8.8–31.1)
≥1.0	0.0 (0.0–0.0)	100.0 (100.0–100.0)	NE	85.7 (82.9–88.8)	85.7 (82.9–88.8)	0.0 (0.0–0.0)

PPV, positive predictive value; NPV, negative predictive value; F1, harmonic mean of precision and recall. Values, including F1, are apparent percentages with 500-bootstrap percentile 95% intervals in parentheses. NE, not estimable at a boundary threshold.

**Figure 6:**
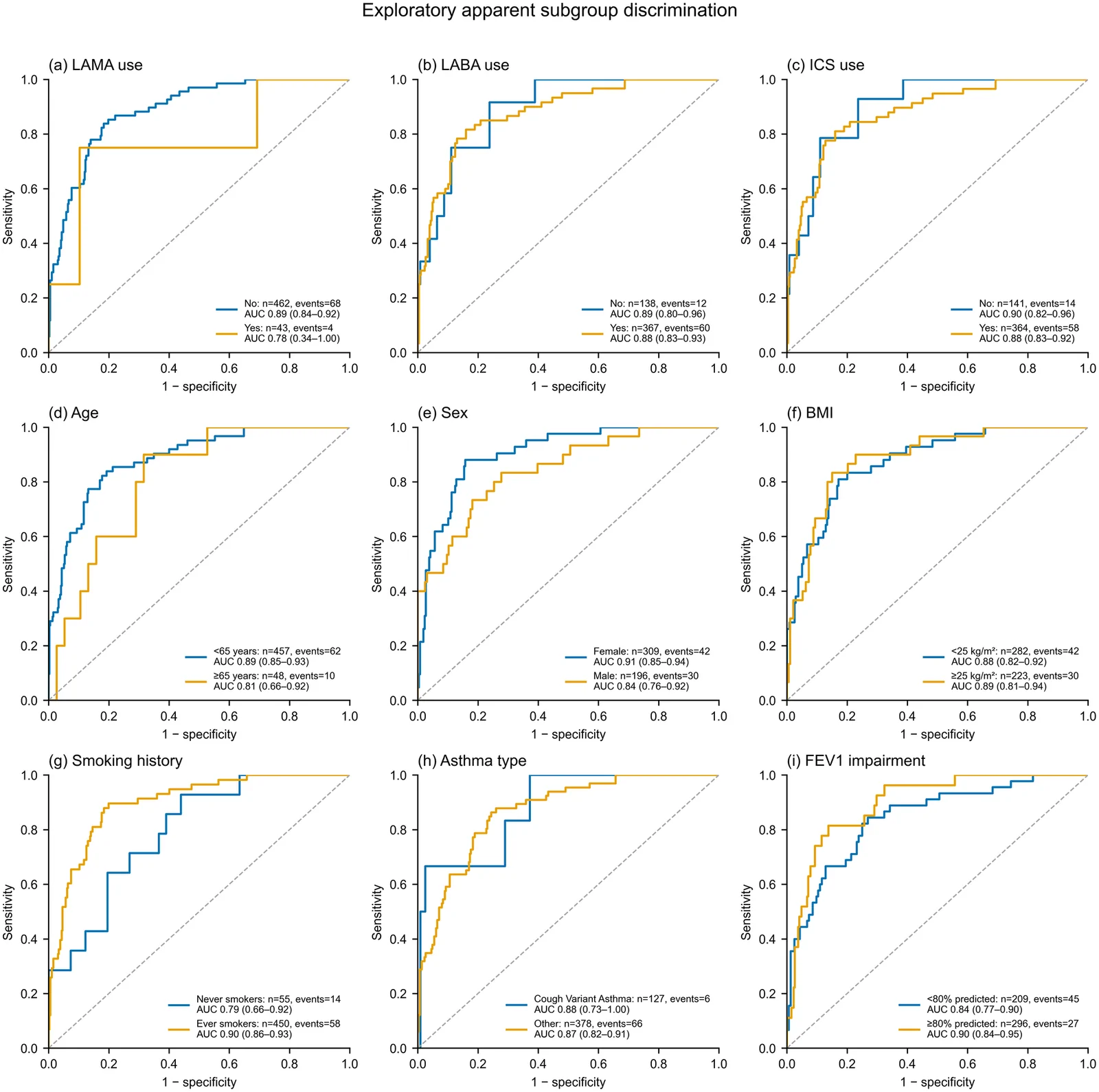
Exploratory apparent subgroup ROC curves from the fixed ten-predictor score. Each legend reports subgroup size, event count, AUC and a conditional percentile 95% interval from 500 patient-bootstrap samples, without model refitting or selection. Small subgroups yield imprecise estimates; no interaction testing was performed. Smoking groups comprise ever smokers (current or former; source Yes) and never smokers (source No).

## 4 Discussion

In this retrospective cohort, the multimodal score incorporated quantitative imaging and clinical information through a static baseline CT digital-twin framework. CT contribution was comparator-dependent: the increment was modest and not statistically significant in the fully nested comparison, whereas the available-variable comparison showed a larger apparent AUC increment. Neither comparison establishes improved out-of-sample performance. Fixed-predictor bootstrap validation was conditional on the selected predictor set, and the model remains a development-stage research tool.

Disease duration was associated with higher exacerbation risk, consistent with the idea that longer-standing asthma may reflect cumulative airway remodeling, treatment burden, or persistent exposure to inflammatory triggers (13). Clinically, this finding reinforces that patients with a longer disease history may remain vulnerable even when a single contemporaneous spirometry measure appears only moderately abnormal.

The pulmonary-function variables contributed to the composite score, but their coefficients should not be interpreted as independent causal effects. TLC% may capture hyperinflation or air trapping, whereas VC%, FVC%, and DLCO% add correlated physiologic information. These interpretations require validation in cohorts with standardized testing and complete clinical severity measures.

IgE and MXD contributed to the fitted score as systemic/allergic inflammatory descriptors. Total IgE is not a complete endotype marker, and MXD represents an aggregate leukocyte-subgroup measure rather than a single cell type. The model does not establish a biological pathway or treatment response.

The two CT vascular variables represent quantitative associations, not demonstrated biological mechanisms. Vascular remodeling, perfusion and histopathology were not directly measured. All baseline scans were noncontrast, and voxel-level review identified no measurement error in the extreme values; maximum venous density nevertheless remains an attenuation descriptor rather than a direct measure of blood flow or perfusion. Its observed range was 117–3,258 HU, with 276/505 values exceeding 500 HU (Supplementary Table S11). Replacing maximum with mean venous density yielded apparent AUC 0.874; this sensitivity analysis does not establish physiological validity.

Contemporary cohorts provide context but not direct validation of this score. SARP and SARP III characterized asthma phenotypes (14, 15), U-BIOPRED linked severe-asthma clusters to inflammatory and sputum-omics profiles (16, 17), and radiomultiomic research replicated quantitative CT clusters and associated them with molecular pathways (18). COPDGene, SPIROMICS and ECLIPSE combined multicenter longitudinal physiology, CT and outcomes (19–22). Fangal and colleagues described overlapping and distinct physiological, transcriptomic and imaging features in smokers with asthma, COPD and asthma–COPD overlap (23). Our cohort lacked comparable molecular profiling and formal overlap-phenotype adjudication; COPD-related physiology may contribute to the selected predictors. Restricting analysis to 434 participants without recorded COPD (54 events) yielded apparent AUC 0.878 (Supplementary Table S11). RESA was developed in severe asthma using prior exacerbations and other clinical predictors, with country-based internal–external validation (12). Our comparator shares only some predictor categories with RESA, uses different medication definitions, and is neither a reconstruction nor an external validation of that published model.

The fixed-predictor bootstrap estimate of AUC was 0.856, compared with apparent AUC 0.880, with corrected calibration slope 0.875, intercept −0.182 and Brier score 0.094. These results omit uncertainty from selecting the original predictors. The alternative Python selection analysis used different tuning and preprocessing details; its mean apparent-versus-original-cohort AUC difference of 0.086 must not be interpreted as exact optimism correction for the original R model.

Digital-twin terminology is not uniform across medical applications. A patient digital-twin scoping review distinguishes static simulation twins used for one-time assessments from dynamic monitoring twins (24), while a chronic-lung-disease review describes the broader role of personalized computational representations (25). Throughout this manuscript, static baseline CT digital-twin framework denotes the baseline patient-specific representation linked to a fixed probability model. The present implementation does not perform mechanistic simulation, dynamic synchronization or bidirectional feedback.

If externally validated, the nomogram could be studied as an aid to risk-informed follow-up, inhaler-technique review, adherence assessment, or treatment discussions. These are hypotheses for prospective evaluation, not demonstrated clinical uses. No prospective impact analysis, treatment-allocation comparison, or evidence that the calculator changes clinician decisions was performed. The web application is therefore a research-use demonstration, not a clinical implementation tool.

This retrospective single-center study is a development study based on the imputed 505-record analysis table; performance estimates are conditional on this analysis table. Selective cohort assembly limits generalizability. The event count and broad candidate set create overfitting risk, and fixed-predictor bootstrap validation does not repeat LASSO selection. Joint segmentation review with 0 manual corrections supports quality control but does not establish inter-reader or scan–rescan measurement reproducibility. The available-variable comparator represents selected clinical domains rather than comprehensive clinical risk assessment. The CT contribution and clinical utility therefore require selection-aware validation, external cohorts, and prospective impact evaluation before implementation.

Future work should validate the original full modeling procedure and assess the score in external cohorts with broader clinical risk domains and prospectively adjudicated outcomes. Repeated segmentation and scan–rescan studies, validation of the physiological meaning of CT features, and prospective impact evaluation are needed. Adding longitudinal imaging, physiological and clinical data, together with a defined updating and feedback mechanism, could extend the static framework toward a conventional dynamic digital twin.

The multimodal score incorporated a static baseline CT digital-twin framework and showed conditional internal performance. CT contribution was comparator-dependent and remained unproven out of sample. Selection-aware validation, quantitative CT repeatability and prospective clinical evaluation are required before implementation.

## Data Availability

The data analyzed in this study is subject to the following licenses/restrictions: the datasets generated and/or analyzed during the current study are not publicly available due to patient privacy restrictions but are available from the corresponding authors on reasonable request. Requests to access these datasets should be directed to Munir251031@163.com.
