# Supplementary material for: Multimodal prediction of documented 1-year asthma exacerbation risk incorporating a static baseline CT digital-twin framework: a retrospective cohort study
Source: Front Digit Health. 2026 Oct 26;8:1935315. doi: 10.3389/fdgth.2026.1935315 (PMC13745687; doi:10.3389/fdgth.2026.1935315)
Supplement: Supplementary file 1 [file Datasheet1.docx]

Table S1. Variables derived from the static baseline CT digital-twin framework (baseline quantitative reconstruction)

| Category | Parameter Name | Unit | Original Identifier |
| --- | --- | --- | --- |
| Both Lungs | Volume | ml | BothLungs Volume ml |
| Both Lungs | LAA < -910HU Volume | ml | BothLungs LAA910 ml |
| Both Lungs | LAA < -950HU Volume | ml | BothLungs LAA950 ml |
| Both Lungs | LAA < -910HU Percentage | % | BothLungs LAA910 percent |
| Both Lungs | LAA < -950HU Percentage | % | BothLungs LAA950 percent |
| Both Lungs | Mean Density | HU | BothLungs Mean Density HU |
| Both Lungs | Standard Deviation of Density | HU | BothLungs Density STD HU |
| Both Lungs | Bronchus Count | count | BothLungs Bronchus Count |
| Both Lungs | Bronchus Length | cm | BothLungs Bronchus Length cm |
| Both Lungs | Bronchus Volume | ml | BothLungs Bronchus Volume ml |
| Both Lungs | Agatston Calcification Score | score | BothLungs Agatston Calcification |
| Both Lungs | Calcification Volume | mm3 | BothLungs Volume Calcification mm3 |
| Both Lungs | Vessel Volume | ml | BothLungs Vessel Volume ml |
| Both Lungs | Lung Tissue Volume | ml | BothLungs Lung Tissue Volume ml |
| Both Lungs | Air Volume | ml | BothLungs Air Volume ml |
| Both Lungs | Mass | g | BothLungs Mass g |
| Both Lungs | Angle 1 | degree | BothLungs Angle 1 |
| Both Lungs | Angle 01 | degree | BothLungs Angle 01 |
| Both Lungs | Bronchus Count V2 | count | BothLungs Bronchus Count v2 |
| Both Lungs | Bronchus Length V2 | cm | BothLungs Bronchus Length cm v2 |
| Both Lungs | Bronchus Volume V2 | ml | BothLungs Bronchus Volume ml v2 |
| Both Lungs | Generation Count | num | BothLungs Generation |
| Both Lungs | Bronchus Count Delta 2mm | count | BothLungs Bronchus CountDlt 2mm |
| Both Lungs | Bronchus Volume Delta 2mm | ml | BothLungs Bronchus Volume mlDlt 2mm |
| Both Lungs | Pi10 (Wall Area at 10mm lumen perimeter) | mm | BothLungs Pi10 mm |
| Both Lungs | Tortuosity | ratio | BothLungs Tortuosity |
| Both Lungs | Fractal Dimension | index | BothLungs Fractal Dimension |
| Both Lungs | -950HU Volume | ml | BothLungs Minus950 Volume ml |
| Both Lungs | -950HU Percent | % | BothLungs Minus950 Percent |
| Both Lungs | -950HU Mean Density | HU | BothLungs Minus950 Mean Density HU |
| Both Lungs | -950HU Heterogeneity | index | BothLungs Minus950 Heterogeneity |
| Right Lung | Volume | ml | RightLung Volume ml |
| Right Lung | LAA < -910HU Volume | ml | RightLung LAA910 ml |
| Right Lung | LAA < -950HU Volume | ml | RightLung LAA950 ml |
| Right Lung | LAA < -910HU Percentage | % | RightLung LAA910 percent |
| Right Lung | LAA < -950HU Percentage | % | RightLung LAA950 percent |
| Right Lung | Mean Density | HU | RightLung Mean Density HU |
| Right Lung | Standard Deviation of Density | HU | RightLung Density STD HU |
| Right Lung | Bronchus Count | count | RightLung Bronchus Count |
| Right Lung | Bronchus Length | cm | RightLung Bronchus Length cm |
| Right Lung | Bronchus Volume | ml | RightLung Bronchus Volume ml |
| Right Lung | Agatston Calcification Score | score | RightLung Agatston Calcification |
| Right Lung | Calcification Volume | mm3 | RightLung Volume Calcification mm3 |
| Right Lung | Vessel Volume | ml | RightLung Vessel Volume ml |
| Right Lung | Lung Tissue Volume | ml | RightLung Lung Tissue Volume ml |
| Right Lung | Air Volume | ml | RightLung Air Volume ml |
| Right Lung | Mass | g | RightLung Mass g |
| Right Lung | Angle 1 | degree | RightLung Angle 1 |
| Right Lung | Angle 01 | degree | RightLung Angle 01 |
| Right Lung | Bronchus Count V2 | count | RightLung Bronchus Count v2 |
| Right Lung | Bronchus Length V2 | cm | RightLung Bronchus Length cm v2 |
| Right Lung | Bronchus Volume V2 | ml | RightLung Bronchus Volume ml v2 |
| Right Lung | Generation Count | num | RightLung Generation |
| Right Lung | -950HU Volume | ml | RightLung Minus950 Volume ml |
| Right Lung | -950HU Percent | % | RightLung Minus950 Percent |
| Right Lung | -950HU Mean Density | HU | RightLung Minus950 Mean Density HU |
| Right Lung | -950HU Heterogeneity | index | RightLung Minus950 Heterogeneity |
| Left Lung | Volume | ml | LeftLung Volume ml |
| Left Lung | LAA < -910HU Volume | ml | LeftLung LAA910 ml |
| Left Lung | LAA < -950HU Volume | ml | LeftLung LAA950 ml |
| Left Lung | LAA < -910HU Percentage | % | LeftLung LAA910 percent |
| Left Lung | LAA < -950HU Percentage | % | LeftLung LAA950 percent |
| Left Lung | Mean Density | HU | LeftLung Mean Density HU |
| Left Lung | Standard Deviation of Density | HU | LeftLung Density STD HU |
| Left Lung | Bronchus Count | count | LeftLung Bronchus Count |
| Left Lung | Bronchus Length | cm | LeftLung Bronchus Length cm |
| Left Lung | Bronchus Volume | ml | LeftLung Bronchus Volume ml |
| Left Lung | Agatston Calcification Score | score | LeftLung Agatston Calcification |
| Left Lung | Calcification Volume | mm3 | LeftLung Volume Calcification mm3 |
| Left Lung | Vessel Volume | ml | LeftLung Vessel Volume ml |
| Left Lung | Lung Tissue Volume | ml | LeftLung Lung Tissue Volume ml |
| Left Lung | Air Volume | ml | LeftLung Air Volume ml |
| Left Lung | Mass | g | LeftLung Mass g |
| Left Lung | Angle 1 | degree | LeftLung Angle 1 |
| Left Lung | Angle 01 | degree | LeftLung Angle 01 |
| Left Lung | Bronchus Count V2 | count | LeftLung Bronchus Count v2 |
| Left Lung | -950HU Volume | ml | LeftLung Minus950 Volume ml |
| Left Lung | -950HU Percent | % | LeftLung Minus950 Percent |
| Left Lung | -950HU Mean Density | HU | LeftLung Minus950 Mean Density HU |
| Left Lung | -950HU Heterogeneity | index | LeftLung Minus950 Heterogeneity |
| Vessels (General) | Total Volume | ml | PulmonaryVessels Volume ml |
| Vessels (General) | Max Density | HU | PulmonaryVessels Max Density HU |
| Vessels (General) | Mean Density | HU | PulmonaryVessels Mean Density HU |
| Vessels (General) | Min Density | HU | PulmonaryVessels Min Density HU |
| Vessels (General) | Branch Count | count | PulmonaryVessels Vessel Branch Count |
| Vessels (General) | Tortuosity | ratio | PulmonaryVessels Tortuosity |
| Vessels (General) | Fractal Dimension | index | PulmonaryVessels Fractal Dimension |
| Vessels (General) | BV5 (Vessels < 5mm2) | ml | PulmonaryVessels BV5 ml |
| Vessels (General) | BV10 (Vessels < 10mm2) | ml | PulmonaryVessels BV10 ml |
| Vessels (General) | BV10+ (Vessels > 10mm2) | ml | PulmonaryVessels BV10 plus ml |
| Artery | Total Volume | ml | Artery Volume ml |
| Artery | Max Density | HU | Artery Max Density HU |
| Artery | Mean Density | HU | Artery Mean Density HU |
| Artery | Min Density | HU | Artery Min Density HU |
| Artery | Branch Count | count | Artery Vessel Branch Count |
| Artery | Tortuosity | ratio | Artery Tortuosity |
| Artery | Fractal Dimension | index | Artery Fractal Dimension |
| Artery | BV5 | ml | Artery BV5 ml |
| Artery | BV10 | ml | Artery BV10 ml |
| Artery | BV10+ | ml | Artery BV10 plus ml |
| Vein | Total Volume | ml | Vein Volume ml |
| Vein | Max Density | HU | Vein Max Density HU |
| Vein | Mean Density | HU | Vein Mean Density HU |
| Vein | Min Density | HU | Vein Min Density HU |
| Vein | Branch Count | count | Vein Vessel Branch Count |
| Vein | Tortuosity | ratio | Vein Tortuosity |
| Vein | Fractal Dimension | index | Vein Fractal Dimension |
| Vein | BV5 | ml | Vein BV5 ml |
| Vein | BV10 | ml | Vein BV10 ml |
| Vein | BV10+ | ml | Vein BV10 plus ml |

**Table S2.** Goodness of Fit

| Deviance | Missing | AIC | BIC | C-statistic | McFadden's R² | Cox & Snell's R² | Nagelkerke's R² |
| --- | --- | --- | --- | --- | --- | --- | --- |
| 287.4058 | 0 | 309.4058 | 355.8759 | 0.8798755 | 0.3052885 | 0.2212754 | 0.3956831 |

Note. AIC: Akaike Information Criterion; BIC: Bayesian Information Criterion.

**Table S3.** Predictive performance of the generalized linear model validated by 5-fold cross-validation (Accuracy and Cohen’s Kappa).

| Accuracy | Kappa | AccuracySD | KappaSD |
| --- | --- | --- | --- |
| 0.8575383 | 0.2926997 | 0.03699713 | 0.1692742 |

Note. SD: Standard Deviation; ROC: Receiver Operating Characteristic (Area Under the Curve); Sens: Sensitivity; Spec: Specificity.

**Table S4.** Discrimination performance of the generalized linear model validated by 5-fold cross-validation (AUC, Sensitivity, and Specificity).

| ROC | Sens | Spec | ROCSD | SensSD | SpecSD |
| --- | --- | --- | --- | --- | --- |
| 0.8560296 | 0.332381 | 0.9560278 | 0.0242855 | 0.0826434 | 0.02251308 |

Note. SD: Standard Deviation; ROC: Receiver Operating Characteristic (Area Under the Curve); Sens: Sensitivity; Spec: Specificity.

**Table S5.** Nested domain-model performance

| Model | Predictor domains | AUC | Refit range (2.5th–97.5th percentiles) | Brier | Refit range (2.5th–97.5th percentiles) | Delta AUC | LR P |
| --- | --- | --- | --- | --- | --- | --- | --- |
| Clinical | Clinical/domain variables defined in Methods | 0.735 | 0.680-0.745 | 0.113 | 0.113-0.119 |  |  |
| Clinical + laboratory | Clinical/domain variables defined in Methods | 0.833 | 0.794-0.832 | 0.094 | 0.095-0.103 | 0.098 | <0.001 |
| Clinical + laboratory + pulmonary function | Clinical/domain variables defined in Methods | 0.880 | 0.837-0.875 | 0.084 | 0.085-0.094 | 0.048 | <0.001 |
| Clinical + laboratory + pulmonary function + CT | Clinical/domain variables defined in Methods | 0.894 | 0.834-0.886 | 0.083 | 0.084-0.094 | 0.013 | 0.070 |

Note. AUC and Brier columns are apparent estimates. The adjacent ranges are 2.5th–97.5th percentiles of performance from 500 bootstrap-trained models evaluated on the original, overlapping cohort. They are descriptive refit distributions, not confidence intervals for the adjacent apparent estimates and not independent test-set performance. Likelihood-ratio tests compare successive nested models.

**Table S6.** Bootstrap optimism-corrected performance of the final ten-predictor score

| Metric | Estimate | Conditional apparent CI or descriptive refit range (see note) |
| --- | --- | --- |
| AUC (apparent) | 0.880 | 0.836–0.917 (2,000 patient-bootstrap samples; fitted predictions fixed) |
| AUC (optimism-corrected) | 0.856 | 0.837-0.877 |
| Calibration intercept (corrected) | -0.182 | -0.621-0.284 |
| Calibration slope (corrected) | 0.875 | 0.674-1.084 |
| Brier score (corrected) | 0.094 | 0.087-0.095 |
| Uniform shrinkage factor | 0.875 | same as corrected slope |

Note. The predictor set was fixed in 1,000 bootstrap refits. Optimism equals bootstrap apparent performance minus performance on the original, overlapping cohort; corrected estimates subtract mean optimism from the original apparent estimate. The refit ranges are 2.5th–97.5th percentiles of original-cohort performance, not confidence intervals for optimism-corrected estimates. Calibration intercept and slope were estimated jointly; this intercept is not an intercept-only calibration-in-the-large estimate. Selection uncertainty is not captured.

**Table S7.** Coding and laboratory-definition audit

| Item | Result | Interpretation |
| --- | --- | --- |
| Analysis cohort | 505 records; 72 events | Complete analysis table analyzed |
| Smoking_Status | Yes 450; No 55 | Yes=1: current or former smoking (ever smokers); No=0: never smoking. The analysis compares ever with never smoking. |
| MXD | 0.05-1.52 x10^9/L | MXD is the combined count of blood leukocyte subgroups other than neutrophils and lymphocytes; values are expressed in ×10^9/L. |
| WBC and differential | WBC 3.25-17.98 x10^9/L | WBC denotes total white blood cell count. MXD is the combined count of blood leukocyte subgroups other than neutrophils and lymphocytes; values are expressed in ×10^9/L. |

**Table S8.** Collinearity assessment of the final ten-predictor score

| Variable | Variance inflation factor |
| --- | --- |
| Disease duration | 1.07 |
| VC% | 5.28 |
| FVC% | 5.05 |
| TLC% | 1.15 |
| DLCO% | 1.14 |
| WBC | 1.55 |
| MXD | 1.53 |
| IgE | 1.06 |
| Artery volume | 1.16 |
| Maximum venous density | 1.09 |

Note. VIFs were calculated on the complete 505-record analysis table. VC% and FVC% showed moderate collinearity; the maximum VIF was 5.28, and no predictor exceeded 10.

**Table S9.** Final unpenalized model equation on original variable scales

| Term | Coefficient | Operational scale |
| --- | --- | --- |
| (Intercept) | -7.365236557 | log-odds |
| Disease duration | 0.067492959 | per year |
| VC% | -0.019554053 | per percentage point |
| FVC% | -0.009019932 | per percentage point |
| TLC% | 0.047070055 | per percentage point |
| DLCO% | -0.010961851 | per percentage point |
| WBC | 0.130042741 | per 1 x10^9/L |
| MXD | 2.706085526 | per 1 x10^9/L |
| IgE | 0.002248868 | per IU/mL |
| Artery volume | 0.004668169 | per mL |
| Maximum venous density | 0.000614886 | per HU |

Predicted risk = expit(intercept + sum coefficient x recorded predictor). No standardization is applied when this equation is used; the equation is a descriptive refit of the analysis table and has not been externally validated.

**Table S10.** Exploratory selection stability using an alternative Python implementation

| Feature | Selection frequency | Selection count |
| --- | --- | --- |
| TLC_percent | 0.998 | 499 |
| IgE | 0.996 | 498 |
| Disease_Duration_Years | 0.982 | 491 |
| MXD | 0.976 | 488 |
| EOS | 0.872 | 436 |
| Drug_OCS_Yes | 0.856 | 428 |
| Drug_LABA_Yes | 0.836 | 418 |
| Comorbidity_Rhinitis_Yes | 0.814 | 407 |
| NEUT | 0.810 | 405 |
| VC_percent | 0.794 | 397 |
| Vein_Max_Density_HU | 0.778 | 389 |
| Smoking_Status_Yes | 0.770 | 385 |
| Diagnosis_Code_Other | 0.736 | 368 |
| Drug_LAMA_Yes | 0.674 | 337 |
| RightLung_Minus950_Heterogeneity | 0.668 | 334 |
| BothLungs_Bronchus_CountDlt_2mm | 0.648 | 324 |
| BothLungs_Tortuosity | 0.636 | 318 |
| DLCO_percent | 0.632 | 316 |
| FVC_percent | 0.574 | 287 |
| Vein_Fractal_Dimension | 0.558 | 279 |

In 500 exploratory resamples, mean bootstrap apparent AUC was 0.963 and mean performance on the original, overlapping cohort was 0.877 (2.5th–97.5th percentile range, 0.840–0.909). The mean difference was 0.086. Python used eight inverse-penalty values from 10^-3 to 10^2, five-fold negative-log-loss tuning with a 1-SE selection rule, liblinear L1 fitting, and an approximately unpenalized refit (C=10^6). Scaling preceded inner cross-validation and bootstrap duplicates were not grouped by patient in the inner folds; liblinear also penalizes the intercept. The platform administrator confirmed agreement in modeling settings, but numerical equivalence to the original R workflow has not been demonstrated. Subtracting 0.086 from the original model AUC is therefore not reported as a valid full-procedure optimism-corrected AUC. Exact validation of the original selection procedure remains unresolved.

**Table S11.** CT distribution and sensitivity analyses

| Analysis | N (events) | AUC | Brier / CT coefficient |
| --- | --- | --- | --- |
| No COPD | 434 (54) | 0.878 | 0.081; OR/100 HU 1.059 |
| Vein max density <=500 HU | 229 (14) | 0.946 | 0.037; OR/100 HU 1.799 |
| Replace maximum venous density with mean venous density | 505 (72) | 0.874 | 0.087; OR/100 HU 1.480 |

Maximum venous density: minimum 117, Q1 307, median 653, Q3 1,073 and maximum 3,258 HU; 276/505 values exceeded 500 HU. All scans were noncontrast, and voxel-level review confirmed the recorded extreme values with no measurement error identified. This verification does not establish physiological specificity or validate maximum attenuation as a perfusion measure.

**Table S12.** Bootstrap uncertainty for apparent threshold metrics

| Threshold | Sensitivity | Specificity | PPV | NPV | Accuracy | F1 |
| --- | --- | --- | --- | --- | --- | --- |
| >=0.0 | 1.000 (1.000-1.000) | 0.000 (0.000-0.000) | 0.143 (0.115-0.171) | NE | 0.143 (0.115-0.171) | 0.250 (0.206-0.293) |
| >=0.1 | 0.861 (0.774-0.937) | 0.762 (0.718-0.800) | 0.376 (0.305-0.443) | 0.971 (0.952-0.987) | 0.776 (0.741-0.813) | 0.523 (0.446-0.595) |
| >=0.2 | 0.611 (0.481-0.720) | 0.896 (0.869-0.925) | 0.494 (0.384-0.600) | 0.933 (0.908-0.956) | 0.855 (0.826-0.883) | 0.547 (0.439-0.646) |
| >=0.3 | 0.528 (0.404-0.639) | 0.938 (0.914-0.960) | 0.585 (0.458-0.709) | 0.923 (0.896-0.944) | 0.879 (0.848-0.907) | 0.555 (0.445-0.653) |
| >=0.4 | 0.417 (0.305-0.533) | 0.952 (0.930-0.970) | 0.588 (0.457-0.720) | 0.907 (0.878-0.934) | 0.875 (0.844-0.903) | 0.488 (0.374-0.592) |
| >=0.5 | 0.361 (0.255-0.473) | 0.958 (0.939-0.977) | 0.591 (0.447-0.744) | 0.900 (0.874-0.929) | 0.873 (0.844-0.903) | 0.448 (0.332-0.559) |
| >=0.6 | 0.306 (0.206-0.415) | 0.984 (0.972-0.995) | 0.759 (0.593-0.906) | 0.895 (0.867-0.921) | 0.887 (0.860-0.913) | 0.436 (0.311-0.549) |
| >=0.7 | 0.264 (0.171-0.375) | 0.995 (0.988-1.000) | 0.905 (0.765-1.000) | 0.890 (0.865-0.918) | 0.891 (0.863-0.917) | 0.409 (0.284-0.537) |
| >=0.8 | 0.167 (0.087-0.261) | 0.995 (0.988-1.000) | 0.857 (0.667-1.000) | 0.878 (0.850-0.905) | 0.877 (0.850-0.903) | 0.279 (0.156-0.408) |
| >=0.9 | 0.111 (0.047-0.190) | 0.995 (0.988-1.000) | 0.800 (0.500-1.000) | 0.871 (0.840-0.898) | 0.869 (0.839-0.896) | 0.195 (0.088-0.311) |
| >=1.0 | 0.000 (0.000-0.000) | 1.000 (1.000-1.000) | NE | 0.857 (0.829-0.888) | 0.857 (0.829-0.888) | 0.000 (0.000–0.000) |

Values are apparent estimates with percentile intervals from 500 bootstrap resamples of the fixed fitted score. PPV and NPV are marked NE (not estimable) when no positive or no negative classifications occur.

**Table S13.** Available conventional comparator and CT increment

| Model | Predictors | N | Events | AUC | Brier |
| --- | --- | --- | --- | --- | --- |
| available conventional comparator | Age;Gender;BMI;Smoking_Status;FEV1_percent;EOS;Drug_ICS;Drug_OCS;Comorbidity_COPD | 505 | 72 | 0.725 | 0.111 |
| available conventional + CT | Age;Gender;BMI;Smoking_Status;FEV1_percent;EOS;Drug_ICS;Drug_OCS;Comorbidity_COPD;Artery_Volume_ml;Vein_Max_Density_HU | 505 | 72 | 0.774 | 0.107 |

The comparator used age, sex (Female=0, Male=1), BMI, ever-smoking status, FEV1 percent predicted, eosinophils, ICS, OCS and COPD. Only some predictor categories overlap with RESA [reference 12]; BMI, smoking, ICS and COPD are additional available clinical covariates, and current OCS coding is not established as long-term OCS use. The model is not a reconstruction of RESA. Adding CT increased apparent AUC from 0.725 to 0.774 (difference 0.049; conditional bootstrap 95% CI, 0.008–0.092; likelihood-ratio chi-square=17.83, 2 df, P<0.001). The Brier improvement was 0.0039 (95% CI, −0.0018 to 0.0095). Paired 2,000-resample intervals hold fitted predictions fixed and do not account for fitting or selection. For the fully nested comparison, the AUC difference was 0.013 (95% CI, −0.001 to 0.031; likelihood-ratio P=0.070). This comparison is limited to the listed predictor domains and does not represent a complete clinical risk assessment.

**Table S14.** Contextual comparison with contemporary multimodal cohorts

| Cohort/study | Population/design | Modalities | Relevance and key difference |
| --- | --- | --- | --- |
| SARP / SARP III | Asthma phenotyping cohorts; cross-sectional and longitudinal components | Clinical, physiology, inflammatory and cluster variables | Established heterogeneity and severity phenotypes; not a CT-only incremental prediction test |
| U-BIOPRED | European severe-asthma cohort with sputum omics | Clinical, inflammatory, sputum transcriptomic/cluster data | Deep airway inflammatory phenotyping unavailable in the present table |
| SPIROMICS | Multicenter COPD longitudinal cohort | Clinical, physiology, CT and outcomes | Multicenter longitudinal design; different target disease and outcome |
| COPDGene / ECLIPSE | Large COPD cohorts with longitudinal CT and clinical data | CT, physiology, clinical and molecular/biomarker data | Demonstrate chronic-airway heterogeneity and protocolized follow-up; not directly transportable to this asthma cohort |
| Zounemat Kermani et al. | Severe-asthma radiomultiomics study | Quantitative CT linked to molecular pathways | Mechanistic/radiomultiomic objective differs from this internally evaluated risk score |
| Fangal et al. | Asthma, COPD and ACO in smokers | Physiology, transcriptomics and imaging | Supports distinct overlap phenotypes; the present cohort lacks comparable molecular data and uses a documented exacerbation outcome |
| Chen et al., RESA | 9,911 severe-asthma patients; international registries | 12-month ≥1/≥2 severe exacerbations; prior exacerbations, control, physiology, biomarkers and treatment | Country-based internal–external validation: pooled time-dependent AUC 0.63 (95% CI 0.60–0.66) for ≥1 and 0.68 (0.64–0.72) for ≥2; not a head-to-head comparison with this cohort |

This table is contextual rather than a head-to-head performance comparison. The present study has no external cohort, molecular measurements, or harmonized longitudinal CT protocol.

**Table S15.** Complete LASSO coefficient path at the selected lambda

| Variable | LASSO coefficient |
| --- | --- |
| (Intercept) | -5.84961988929 |
| Age | 0.00000000000 |
| Gender Male | 0.00000000000 |
| Weight kg | 0.00000000000 |
| Height cm | 0.00000000000 |
| BMI | 0.00000000000 |
| Smoking Status Yes | 0.00000000000 |
| Diagnosis Code Other | 0.00000000000 |
| Disease Duration Years | 0.04002332850 |
| VC percent | -0.00297878502 |
| FVC percent | -0.00417291507 |
| FEV1 percent | 0.00000000000 |
| FEV1 FVC | 0.00000000000 |
| PEF percent | 0.00000000000 |
| TLC percent | 0.02641285646 |
| DLCO percent | -0.00196607009 |
| Drug ICS Yes | 0.00000000000 |
| Drug LABA Yes | 0.00000000000 |
| Drug LAMA Yes | 0.00000000000 |
| Drug SABA Yes | 0.00000000000 |
| Drug LTRA Yes | 0.00000000000 |
| Drug Xanthines Yes | 0.00000000000 |
| Drug OCS Yes | 0.00000000000 |
| Drug AntiHistamines Yes | 0.00000000000 |
| Drug CompoundMethoxyphenamine Yes | 0.00000000000 |
| Comorbidity Rhinitis Yes | 0.00000000000 |
| Comorbidity COPD Yes | 0.00000000000 |
| Comorbidity Polyp Sinusitis Yes | 0.00000000000 |
| WBC | 0.06703868185 |
| NEUT | 0.00000000000 |
| LYM | 0.00000000000 |
| MXD | 1.91750919618 |
| EOS | 0.00000000000 |
| BASO | 0.00000000000 |
| IgE | 0.00115869285 |
| BothLungs Volume ml | 0.00000000000 |
| BothLungs LAA910 ml | 0.00000000000 |
| BothLungs LAA950 ml | 0.00000000000 |
| BothLungs LAA910 percent | 0.00000000000 |
| BothLungs LAA950 percent | 0.00000000000 |
| BothLungs Mean Density HU | 0.00000000000 |
| BothLungs Bronchus Count | 0.00000000000 |
| BothLungs Bronchus Length cm | 0.00000000000 |
| BothLungs Bronchus Volume ml | 0.00000000000 |
| BothLungs Agatston Calcification | 0.00000000000 |
| BothLungs Volume Calcification mm3 | 0.00000000000 |
| BothLungs Vessel Volume ml | 0.00000000000 |
| BothLungs Lung Tissue Volume ml | 0.00000000000 |
| BothLungs Air Volume ml | 0.00000000000 |
| BothLungs Density STD HU | 0.00000000000 |
| BothLungs Mass g | 0.00000000000 |
| BothLungs Angle 1 | 0.00000000000 |
| BothLungs Angle 01 | 0.00000000000 |
| RightLung Volume ml | 0.00000000000 |
| RightLung LAA910 ml | 0.00000000000 |
| RightLung LAA950 ml | 0.00000000000 |
| RightLung LAA910 percent | 0.00000000000 |
| RightLung LAA950 percent | 0.00000000000 |
| RightLung Mean Density HU | 0.00000000000 |
| RightLung Bronchus Count | 0.00000000000 |
| RightLung Bronchus Length cm | 0.00000000000 |
| RightLung Bronchus Volume ml | 0.00000000000 |
| RightLung Agatston Calcification | 0.00000000000 |
| RightLung Volume Calcification mm3 | 0.00000000000 |
| RightLung Vessel Volume ml | 0.00000000000 |
| RightLung Lung Tissue Volume ml | 0.00000000000 |
| RightLung Air Volume ml | 0.00000000000 |
| RightLung Density STD HU | 0.00000000000 |
| RightLung Mass g | 0.00000000000 |
| RightLung Angle 1 | 0.00000000000 |
| RightLung Angle 01 | 0.00000000000 |
| LeftLung Volume ml | 0.00000000000 |
| LeftLung LAA910 ml | 0.00000000000 |
| LeftLung LAA950 ml | 0.00000000000 |
| LeftLung LAA910 percent | 0.00000000000 |
| LeftLung LAA950 percent | 0.00000000000 |
| LeftLung Mean Density HU | 0.00000000000 |
| LeftLung Bronchus Count | 0.00000000000 |
| LeftLung Bronchus Length cm | 0.00000000000 |
| LeftLung Bronchus Volume ml | 0.00000000000 |
| LeftLung Agatston Calcification | 0.00000000000 |
| LeftLung Volume Calcification mm3 | 0.00000000000 |
| LeftLung Vessel Volume ml | 0.00000000000 |
| LeftLung Lung Tissue Volume ml | 0.00000000000 |
| LeftLung Air Volume ml | 0.00000000000 |
| LeftLung Density STD HU | 0.00000000000 |
| LeftLung Mass g | 0.00000000000 |
| LeftLung Angle 1 | 0.00000000000 |
| LeftLung Angle 01 | 0.00000000000 |
| BothLungs Bronchus Count v2 | 0.00000000000 |
| BothLungs Bronchus Length cm v2 | 0.00000000000 |
| BothLungs Bronchus Volume ml v2 | 0.00000000000 |
| BothLungs Generation | 0.00000000000 |
| BothLungs Bronchus CountDlt 2mm | 0.00000000000 |
| BothLungs Bronchus Volume mlDlt 2mm | 0.00000000000 |
| BothLungs Pi10 mm | 0.00000000000 |
| BothLungs Tortuosity | 0.00000000000 |
| BothLungs Fractal Dimension | 0.00000000000 |
| RightLung Bronchus Count v2 | 0.00000000000 |
| RightLung Bronchus Length cm v2 | 0.00000000000 |
| RightLung Bronchus Volume ml v2 | 0.00000000000 |
| RightLung Generation | 0.00000000000 |
| LeftLung Bronchus Count v2 | 0.00000000000 |
| PulmonaryVessels Volume ml | 0.00000000000 |
| PulmonaryVessels Max Density HU | 0.00000000000 |
| PulmonaryVessels Mean Density HU | 0.00000000000 |
| PulmonaryVessels Min Density HU | 0.00000000000 |
| PulmonaryVessels Vessel Branch Count | 0.00000000000 |
| PulmonaryVessels Tortuosity | 0.00000000000 |
| PulmonaryVessels Fractal Dimension | 0.00000000000 |
| PulmonaryVessels BV5 ml | 0.00000000000 |
| PulmonaryVessels BV10 ml | 0.00000000000 |
| PulmonaryVessels BV10 plus ml | 0.00000000000 |
| Artery Volume ml | 0.00060838793 |
| Artery Max Density HU | 0.00000000000 |
| Artery Mean Density HU | 0.00000000000 |
| Artery Min Density HU | 0.00000000000 |
| Artery Vessel Branch Count | 0.00000000000 |
| Artery Tortuosity | 0.00000000000 |
| Artery Fractal Dimension | 0.00000000000 |
| Artery BV5 ml | 0.00000000000 |
| Artery BV10 ml | 0.00000000000 |
| Artery BV10 plus ml | 0.00000000000 |
| Vein Volume ml | 0.00000000000 |
| Vein Max Density HU | 0.00008693638 |
| Vein Mean Density HU | 0.00000000000 |
| Vein Min Density HU | 0.00000000000 |
| Vein Vessel Branch Count | 0.00000000000 |
| Vein Tortuosity | 0.00000000000 |
| Vein Fractal Dimension | 0.00000000000 |
| Vein BV5 ml | 0.00000000000 |
| Vein BV10 ml | 0.00000000000 |
| Vein BV10 plus ml | 0.00000000000 |
| BothLungs Minus950 Volume ml | 0.00000000000 |
| BothLungs Minus950 Percent | 0.00000000000 |
| BothLungs Minus950 Mean Density HU | 0.00000000000 |
| BothLungs Minus950 Heterogeneity | 0.00000000000 |
| RightLung Minus950 Volume ml | 0.00000000000 |
| RightLung Minus950 Percent | 0.00000000000 |
| RightLung Minus950 Mean Density HU | 0.00000000000 |
| RightLung Minus950 Heterogeneity | 0.00000000000 |
| LeftLung Minus950 Volume ml | 0.00000000000 |
| LeftLung Minus950 Percent | 0.00000000000 |
| LeftLung Minus950 Mean Density HU | 0.00000000000 |
| LeftLung Minus950 Heterogeneity | 0.00000000000 |

This table preserves the complete coefficient vector, including zero coefficients, for transparent reporting of the feature-selection step. The final unpenalized equation is reported separately in Supplementary Table S9.

**Supplementary Methods S1. Python reanalysis specification**

The 505-participant analysis table and 72 outcome events were retained without additional imputation or case deletion. The original R 4.2.2 model was generated automatically by MSTAT. The authors confirmed with the MSTAT platform administrator that the modeling parameter settings matched those documented for the Python selection implementation in Supplementary Methods S1. The fixed-predictor bootstrap is conditional on the ten selected predictors and does not repeat LASSO tuning or variable selection; the supplementary bootstrap and comparison procedures are additional analyses.

Nested-domain and fixed-predictor bootstrap models used scikit-learn logistic regression with the lbfgs solver, inverse regularization strength C=10^6 and max_iter=3000. Numerical predictors were standardized within each fitted sample; categorical predictors used one-hot encoding with the first level omitted and unknown categories ignored. Median/mode imputation steps in the pipeline had no missing values to replace in this complete analysis table. The ten-predictor final equation and available-variable comparator were also fitted as binomial generalized linear models with a logit link using statsmodels on the recorded scales (Table S9). Sex was coded Female=0/Male=1 and binary Yes/No fields as No=0/Yes=1. Nested likelihood-ratio tests used binomial GLMs on the same design matrices.

Fixed-predictor optimism correction used 1,000 patient-level bootstrap samples, NumPy default_rng seed 20260909, with preprocessing and model refitting repeated while the predictor set remained fixed. Performance in each bootstrap sample was compared with performance of that fitted model in the original, overlapping cohort. Mean optimism was subtracted from the corresponding apparent estimate. Calibration regressed outcome on the logit of probabilities clipped to [10^-6, 1-10^-6], jointly estimating intercept and slope. The 500 refits per nested-domain model used seed 20260910; their percentile ranges describe refit performance, not confidence intervals for optimism-corrected performance.

Paired CT-increment intervals used 2,000 patient-resampling replicates with predictions held fixed and seed 20260916. The complete nested comparison and the sex-corrected available-variable comparison were generated separately. Percentile endpoints were the 2.5th and 97.5th percentiles. Brier improvement was reduced-model Brier minus CT-extended-model Brier. These are conditional apparent-performance intervals and omit model-fitting and selection uncertainty.

Threshold intervals used 500 fixed-prediction patient resamples for each threshold from 0 to 1 in increments of 0.1, with seed 20260911. Subgroup AUC intervals used 500 fixed-prediction resamples per subgroup, discarding replicates with only one outcome class, with seed 20260921. The final-score apparent AUC interval used 2,000 additional resamples from the same random-number stream after the subgroup analyses. These intervals do not measure between-subgroup interaction or independent validation.

The exploratory alternative selection analysis used 500 patient bootstrap samples (seed 20260912). Categorical predictors were one-hot encoded with the first level omitted; all encoded columns were standardized within the outer bootstrap sample. L1 logistic regression used liblinear, max_iter=1000, eight C values equally spaced on a log10 scale from 10^-3 to 10^2, and five-fold negative-log-loss tuning (random_state=20260911). The smallest C with mean score within one standard error of the best mean score was chosen; nonzero coefficients were defined by absolute magnitude >10^-8. If no coefficient exceeded the threshold, the feature with the largest absolute coefficient was retained. Selected variables were refitted using lbfgs, C=10^6 and max_iter=3000. Scaling preceded inner cross-validation, bootstrap duplicates were not grouped in inner folds, and liblinear penalized the intercept; these limitations preclude interpreting the analysis as exact validation of the MSTAT selection workflow.

A reproducibility environment checked for the final equation and clinical comparator comprised Python 3.14.3, NumPy 2.4.6, pandas 2.3.3, SciPy 1.17.1, scikit-learn 1.9.0 and statsmodels 0.14.6. This environment description applies to the Python implementation, not the original MSTAT platform. The environment description applies to the Python implementation; the original MSTAT model remains the reference implementation.
